# Molecular Analysis of Methanogen Richness in Landfill and Marshland Targeting 16S rDNA Sequences

**DOI:** 10.1155/2015/563414

**Published:** 2015-10-13

**Authors:** Shailendra Yadav, Sharbadeb Kundu, Sankar K. Ghosh, S. S. Maitra

**Affiliations:** ^1^Department of Biotechnology, Assam University, Silchar, Assam 788011, India; ^2^School of Biotechnology, Jawaharlal Nehru University, New Delhi 110067, India

## Abstract

Methanogens, a key contributor in global carbon cycling, methane emission, and alternative energy production, generate methane gas via anaerobic digestion of organic matter. The methane emission potential depends upon methanogenic diversity and activity. Since they are anaerobes and difficult to isolate and culture, their diversity present in the landfill sites of Delhi and marshlands of Southern Assam, India, was analyzed using molecular techniques like 16S rDNA sequencing, DGGE, and qPCR. The sequencing results indicated the presence of methanogens belonging to the seventh order and also the order Methanomicrobiales in the Ghazipur and Bhalsawa landfill sites of Delhi. Sequences, related to the phyla Crenarchaeota (thermophilic) and Thaumarchaeota (mesophilic), were detected from marshland sites of Southern Assam, India. Jaccard analysis of DGGE gel using Gel2K showed three main clusters depending on the number and similarity of band patterns. The copy number analysis of hydrogenotrophic methanogens using qPCR indicates higher abundance in landfill sites of Delhi as compared to the marshlands of Southern Assam. The knowledge about “methanogenic archaea composition” and “abundance” in the contrasting ecosystems like “landfill” and “marshland” may reorient our understanding of the Archaea inhabitants. This study could shed light on the relationship between methane-dynamics and the global warming process.

## 1. Introduction

Methane is an important greenhouse gas because it is 25 times more powerful than CO_2_ in global warming potential (i.e., the ability of the gas to trap heat in the atmosphere) and thus plays a crucial role in climate change and carbon cycling [1, 2]. Methane emission has contributed approximately 20% to global climate change from preindustrial times [1, 3]. About 500–600 Tg of methane is emitted annually to the atmosphere of which 74% is biogenic, produced by methanogenic Archaea [4].

The methanogenic Archaea (methanogens) usually occurs in highly reduced, anoxic environments such as landfills, wetlands, rice fields, rumen, and marine sediments where they serve as a terminal electron sink [5, 6]. Methanogens are strict anaerobes and the presence of oxygen leads to the formation of reactive oxygen species (ROS), which damage their cell membranes, DNA, and proteins [7, 8]. Methanogens are phylogenetically divided into 5 families within the phylum Euryarchaeota and are comprised of 31 known genera [9, 10]. Methanogens can utilize a wide range of compounds for methane production, but, in most natural systems, there are two major pathways for methanogenesis, reduction of CO_2_ (hydrogenotrophic methanogenesis) and cleavage of acetates (acetoclastic methanogenesis). A third pathway for methane generation is called methylotrophic methanogenesis that occurs in marine sediments and salt lakes where methane is produced from methylated compounds such as trimethylamine [11, 12].

Landfill sites are the third largest source of methane. It constitutes about 30 and 24% of the anthropogenic methane production in Europe and US, respectively [4, 13]. In comparison to the western countries, the composition of municipal solid waste (MSW) in developing countries like India is higher (40–60%) in organic waste. This has more potential to emit higher GHGs (Green House Gases) per ton of MSW compared to the developed world [14]. Moreover, landfills in India are neither well planned nor engineered and are often found in low-lying open areas, where municipal waste is haphazardly and indiscriminately disposed. These sites have neither landfill lining to avoid percolation of leachate to groundwater table nor leachate collection facility. The city generates about 6000 tonnes of solid waste per day and the expected quantity of solid waste generation in Delhi would be about 12,750 tonnes per day by 2015 [15]. Due to scarcity of land in big cities, municipal authorities are using the same landfill for nearly 10–20 years. Thus, the possibility of anaerobic emission of GHGs further increases [16].

Microbial decomposition, climatic conditions, MSW wastes characteristics, and landfilling operations are among the many factors that contribute to the generation of methane [2, 17]. The migration of gas and leachate away from the landfill boundaries and their release into the surrounding environment present serious environmental threats, including potential health hazards, fires and explosions, damage to vegetation, unpleasant odors, landfill settlement, ground water pollution, air pollution, and global warming [18–20].

Wetlands (marshland) are the largest source of natural methane emissions contributing about 10–231 Tg methane per year accounting for 20–39% of annual global CH_4_ emission [4, 21]. Methanogens in the moist, anoxic (oxygen-free) wetland soil produce CH_4_ as they decompose dead plant material. The methane emission from wetland was increased by 7% from 2003 to 2007 [2, 19]. Methane production in wetlands is affected by the acetate supply through acetate fermentation or the CO_2_ reduction potential [22, 23]. The exponential increase in the rate of CH_4_ production with temperature is due to the availability of more substrates and is not associated with changes in the composition of methanogens [24]. Methanogens belonging to the groups Methanomicrobiales and Methanosarcinales performing acetoclastic and methylotrophic pathway were found to be dominant in landfill sites [25–27]. In acidic conditions, due to the presence of acid tolerant hydrogenotrophic methanogens, H_2_/CO_2_ is efficiently converted to methane compared to acetate, and methanogenic activity decreases with decrease in pH regardless of the substrates [28].

The prokaryotic diversity in our planet dictates our planet's ecosystems by acting as key functional drivers [29]. The understanding of the functional potential of the most individual microbial flora residing within the ecosystem is extremely limited because of our inability to isolate and culture them in laboratory conditions [30]. Since the methanogens are anaerobes and are difficult to culture, they are identified by culture independent molecular techniques like PCR amplification, denaturing gradient gel electrophoresis (DGGE), and quantitative real-time PCR, using molecular markers such as 16S rDNA genetic locus [31–34]. Hence, the present study was aimed at detecting the methanogenic Archaea inhabitants (richness) (by DGGE), identification by DNA sequencing, and quantification by qPCR in both the landfill sites of Delhi and marshland sites of Southern Assam, India.

## 2. Material and Methods

### 2.1. Collection of Leachate and Sediment Samples

Leachate samples were collected from three landfill sites (Bhalswa, Okhla, and Ghazipur) in the area of New Delhi, India. These sites are active landfill sites and are still in use. They do not have the leachate collection facility or landfill liner to avoid percolation of leachate to the ground water table (aquifer). Soil, sediment sample was collected from marshlands (Silcoorie Lake (Silchar), Badarpur, and Karimganj) of Southern Assam, India, in sterile falcon tubes. The details of sites along with criteria and physiochemical parameters are shown in Tables 1 and 2.

### 2.2. Nucleic Acid Extraction, PCR Amplification, and Cloning

DNA from both landfill leachate and marshland sediment samples was extracted on the same day of sampling using Fast DNA Spin Kit for Soil (MP Biomedicals, CA, USA). DNA from the marshlands and landfill leachate was amplified using the primer set 86FWD and 1340REV (Table 3).

The amplification profile was 94°C for 5 min, 94°C for 30 s for 30 cycles, and 58°C for 1 minute, elongation at 72°C for 2 minutes, and final extension at 72°C for 10 minutes followed by a cooling step down to 4°C [35, 36]. Obtained 16S rDNA PCR products were purified by PCR purification kit (Fermentas, UK) as recommended by manufacturer protocol. PCR amplicons of 16S rDNA gene were cloned inside PTZ57R/T vector using the Insta-T/A cloning kit (Fermentas, UK) and transformed into* Escherichia coli* DH5*α*. The positive clones were selected using blue-white screening on Luria-Bertani plates containing Ampicillin (100 mg/mL), X-gal (20 mg/mL), and IPTG (100 mM). Then, positive clones were sequenced using M13 FWD primer.

### 2.3. DNA Sequencing and Phylogenetic Analysis of 16S rDNA Clones

Sequencing was performed for all the clones with the ABI prism 3130 Genetic Analyzer (Applied Biosystem Inc., CA) at Department of Biochemistry, South Campus, Delhi University. The sequences were edited to exclude the PCR primer-binding site and manually corrected with Sequence Scanner 1.0 (Applied Biosystems) and were checked further for vector contamination using the Vecscreen tool (http://www.ncbi.nlm.nih.gov/tools/vecscreen/). The sequences showing similarity with vector sequences from both ends were trimmed. Sequences were then compared with the available nucleotide database from the NCBI GenBank using the BLAST program [39]. The partial nucleotide sequences of 16S rDNA genes were submitted to NCBI under accession numbers KM041239 to KM041252 (Table 4).

**Figure 1:**
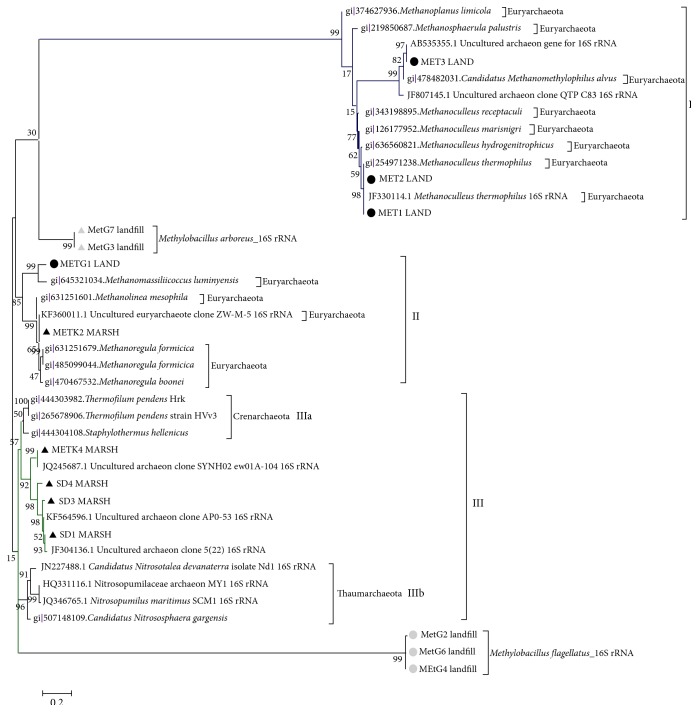
The phylogenetic relationship of 40 partial 16S rDNA sequences (the confirmed 14 sequences of clones are generated in this study, recovered from both Delhi landfills (marked with black circle, grey circle, and grey triangle) and Southern Assam marshland sites (marked with black triangle)) was inferred by the ML method using K2P+G parameter model with 2000 bootstrap replicates using the MEGA 6 tree building program.

Partial 16S rDNA sequences obtained from this study were used for similarity search in NCBI database using BLAST program. After performing BLAST, sequences showing similarity above 90% were used and aligned in MEGA software version 6.0 [40] using ClustalW. The phylogenetic relatedness among clones was estimated using the Maximum Likelihood tree using Kimura K2P+G model with 2000 bootstrap value [41]. For model selection Bayesian analysis was performed and the model with lowest BIC value (i.e., 12104.8604) was chosen for tree construction. All positions containing gaps and missing data were eliminated from the dataset (complete deletion option). The phylogenetic analysis was carried out using MEGA software version 6.0 [40] (Figure 1).

### 2.4. Denaturing Gradient Gel Electrophoresis

**Figure 2:**
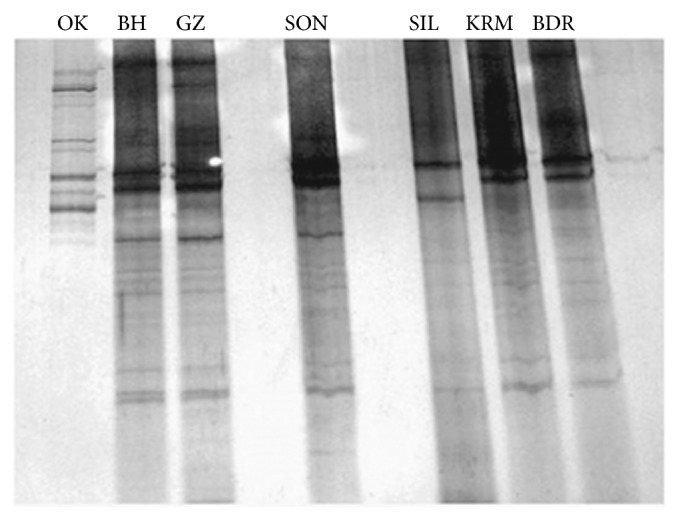
Community profiling of methanogens using 16s rDNA based on DGGE: the community profiling of methanogenic diversity present in the leachate sample of Delhi landfill site (OK, BH, and GZ) and marshland sample of Southern Assam (SON, SIL, KRM, and BDR).

For denaturing gradient gel electrophoresis genomic DNA extracted from landfill and marshland was amplified using primer 519FWD and 915GC which gave a product length of about 500 bp. DGGE was performed with a D-Code universal mutation detection system (Biorad, Hercules, CA, USA) using 16 cm by 16 cm and one mm gels. PCR products were loaded onto 7% (w/v) polyacrylamide gel. The polyacrylamide gels (Bis-Acrylamide, 37.5 : 1) were made with denaturing gradients ranging from 30 to 70%. 100% denaturant contained 7 M urea and 40% formamide. Electrophoresis was initially run at 200 V for 10 min at 60°C and afterwards for 15 h at 85 V. After electrophoresis, the gel was silver-stained and scanned under white light using Gel Doc (Biorad) (Figure 2). DGGE gel was further analyzed using Gel2K software (Svein Norland, Department of Biology, University of Bergen, Norway).

### 2.5. Quantification of Methanogens by Quantitative Real-Time PCR Analysis

Real-time PCR was done for absolute quantification of methanogens. 16S rDNA fragments obtained from pure culture of methanogens (DSMZ) were cloned and serially diluted for making standard curve as was previously done by Steinberg and Regan [27]. Real-time PCR reaction was carried out in triplicate using the temperature profile as recommended for the Agilent 2x master mix, that is, initial denaturation at 95°C for 3 minutes, subsequent denaturation at 95°C for 30 seconds, annealing at 60°C for 10 seconds, and elongation at 65°C for 1 minute. Melt curve analysis to detect the presence of primer dimer was performed after the final extension by increasing the temperature from 50 to 95°C with 0.5°C increments every 10 s.

## 3. Results and Discussions

### 3.1. Identification of Methanogenic Archaea in Landfill and Marshland

Sequences of MET1 LAND and MET2 LAND obtained from the Bhalswa landfill site are clustered with* Methanoculleus thermophilus* methanogens belonging to the order Methanomicrobiales which are hydrogenotrophic in nature. Third sequence of MET3 LAND from the Bhalswa landfill site clustered with the* Candidatus Methanomethylophilus alvus Mx1201*, which is H_2_-dependent methylotrophic methanogens. In Figure 1, it is shown that these three sequences from the landfill sites of Delhi are clustered with Euryarchaeota cluster (Cluster I). Sequence METG1 LAND obtained from the Ghazipur landfill site, Delhi, clustered with* Methanomassiliicoccus luminyensis* (Cluster II). Sequences obtained from marshland sites of Southern Assam were clustered (Cluster III) separately with Crenarchaeota (Cluster IIIa) and Thaumarchaeota (Cluster IIIb). There are five more sequences from the landfill sites of Delhi. They are related to two different species of methanotrophs (methane oxidizing bacteria) (see Table 4),* Methylobacillus arboreus* (marked as grey triangle) and* Methylobacillus flagellatus* (marked as grey circle), and are clustered separately, as shown in Figure 1.

Phylogenetic analysis of 16S rDNA clones indicates the presence of methanogens belonging to the phylum Euryarchaeota, order Methanomicrobiales, Methanobacteriales-1, and seventh order of methanogens in the landfill sites [42–44]. Both* Candidatus Methanomethylophilus alvus Mx1201* and* Methanomassiliicoccus luminyensis* represent a monophyletic lineage that is not phylogenetically associated with any of the previously known orders of methanogens or the anaerobic methanotrophic ANME1 lineage [43, 45]. They belong to the Mx order clusters with two lineages: the planktonic Marine Group II (MG-II) and the sediment dwelling Marine Benthic Group D (MBG-D) [45–47]. The other five sequences from Ghazipur landfill sites revealed presence of methanotrophs belonging to class* Betaproteobacteria*, family Methylophilaceae. 16S rDNA clones obtained from marshland sites of Southern Assam revealed a cluster of Archaea that are distantly related to two different phyla, Crenarchaeota and Thaumarchaeota. Microorganisms belonging to the phylum Thaumarchaeota (recently proposed) are thermophilic and mesophilic in nature and are found to be present in a wide variety of ecosystems, including marine and fresh waters, soils, and also hot environment [44, 48–52].

### 3.2. Culture Independent Molecular Analysis of Methanogenic Diversity

Microbes dominated in the history of living organisms and they are a fundamental part of the biosphere. The study of microbial diversity has been, therefore, essential for understanding the evolution of life. Traditionally, cultivation based methods have contributed to our knowledge about their whereabouts and diversity of microbes in naturally occurring communities. However, only a small fraction of the prokaryotes has been cultivated* in vitro* by standard methods. Therefore, this knowledge may not reveal the actual composition and/or diversity associated with an ecosystem [31, 33]. In the present study, we used culture independent molecular techniques like 16S rDNA PCR, cloning-sequencing, DGGE, and qPCR for estimation of the richness and diversity of the methanogenic Archaea in the landfill site of Delhi and marshland areas of Southern Assam. These techniques are widely used for molecular community analysis of microbes present in various types of habitats [32, 42, 53–56]. A combination of DNA sequencing, DGGE, and quantitative PCR (qPCR) can provide valuable information about microbial consortia associated with a specific ecosystem. Denaturing gradient gel electrophoresis (DGGE) is used to determine the genetic diversity of microbial communities. The procedure is based on electrophoresis of PCR-amplified 16S rDNA fragments in polyacrylamide gels containing a linearly increasing gradient of denaturants. In DGGE, DNA fragments of the same length but with different base-pair composition can be separated. Separation is based on the electrophoretic mobility of partially melted DNA molecules in a polyacrylamide gel and resulting into a band pattern [57–60]. DGGE can reveal 1-2% of the actual diversity present in the samples [61].

### 3.3. Estimation of Methanogenic Richness by Quantitative Real-Time PCR

DNA extracted from the three sampling points, that is, two landfill sites Okhla and Bhalswa of Delhi and Silcoorie Lake (Silchar) of Southern Assam, was screened for the quantification of methanogens. The copy number of all methanogens (pure culture) was higher in the two landfill sites than that of marshland in Southern Assam (Table 5). Methanogenic pathway associated with the methanogens order and its reactions involved in the process are included in Table 6.

The copy numbers of* Methanomicrobium mobile* belonging to the order Methanomicrobiales and* Methanobacterium bryantii* belonging to the order Methanobacteriales-1 were found to be higher in both landfill sites in comparison to the Silcoorie Lake (Silchar) of Southern Assam. Copy number of* Methanobrevibacter arboriphilus* (order Methanobacteriales-1) and* Methanosarcina mazei* (acetoclastic) (order Methanosarcinales) was found to be higher in the Bhalswa landfill site than Okhla landfill site and Silcoorie Lake (Silchar) marshland site. The value of *R*_Sq_ and slope *dR* for standard curve was 0.948 and −2.641, and the efficiency of the reaction was 139.1%. The *R*_Sq_ and slope *dR* values for “absolute” quantification of* Methanobrevibacter arboriphilus* are 0.903 and −2.128. *R*_Sq_ (*dR*) and slope *dR* values for this quantification of* Methanobacterium bryantii* are 0.877 and −1.384. *R*_Sq_ (*dR*) and slope *dR* values of* Methanomicrobium mobile* are 0.956 and −2.563, respectively. The values of *R*_Sq_ (*dR*) and slope *dR* for* Methanosarcina mazei* were 0.394 and −2.051, respectively.

Methanogens pertaining to both acetotrophic and hydrogenotrophic decomposition pathways were detected in MSW landfills, which have been reported earlier [25, 26, 42]. Acetate serves as a precursor for more than 70% of CH_4_ (methane) formation in the most anaerobic digestion process [62]. Therefore, acetoclastic methanogens, which utilize acetate as substrate, play a key role in stabilizing the pollution load of wastewater by methanogenesis. In the present study, quantitative PCR indicates the higher methanogenic richness in both landfill sites of Delhi compared to marshland of Silcoorie Lake, Silchar.

### 3.4. Diversity of Methanogenic Archaea by Denaturing Gradient Gel Electrophoresis

Abundance and diversity of methanogenic Archaea were studied in three landfill and four marshland sites situated at different location in Delhi and Southern Assam, India. 16S rDNA amplicons were cloned and then analyzed on the DGGE gel for estimation of the archaeal richness in respective samples as shown in Figure 2.

**Figure 3:**
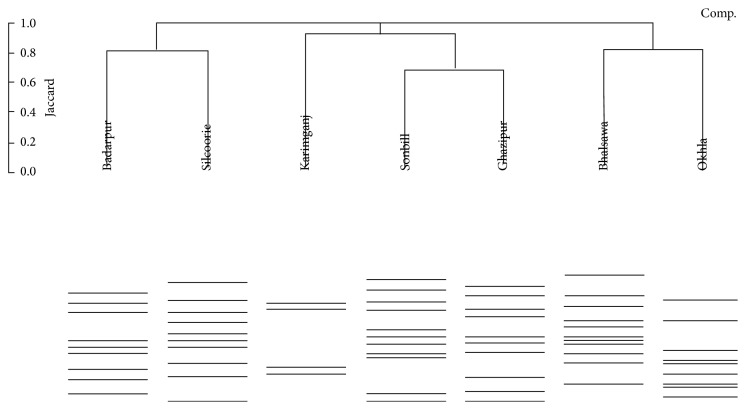
Jaccard cluster diagram of DGGE bands obtained from landfill sites of Delhi and marshland of Southern Assam, India.

Band patterns of 16S rDNA amplicons obtained from the landfill sites (OK, BH, and GZ) of Delhi and marshland samples (SON, SIL, KRM, and BDR) of Southern Assam were compared for methanogens richness and diversity analysis using Gel2K software. Analysis of DGGE image revealed the presence of total 38 bands. There are some unique bands in each lane, which indicates the variation of methanogens community residing in those particular samples. Cluster analysis of bands using Jaccard analysis indicated the presence of three main clusters consisting of localities that differ in number of similarity versus DGGE bands (Figure 3).

In the first cluster, Badarpur beetle-nut pond and Silcoorie Lake (Silchar) of Southern Assam clustered together showing similar band pattern. In the second cluster, interestingly, despite being two different ecosystems, Ghazipur landfill sites of Delhi clustered with wetland of Sonbill, Southern Assam, India. In the third cluster, the two landfill sites of Delhi (Okhla and Bhalswa) clustered together showing similar band pattern. In terms of richness, number of bands from the respective samples from Bhalswa landfill and Sonbill wetland have maximum of 11 bands, followed by Ghazipur landfill site and Silcoorie Lake (Silchar) having 10 bands each. Okhla landfill and Badarpur beetle-nut pond showed 9 bands each in the cluster. In the Karimganj rice paddy field sample, only four bands were observed showing the least diversity.

Microbial diversity within contaminated ecosystems like landfill should be less diverse than those in natural systems like a wetland because the diversity may be influenced by the complexity of toxic chemical mixtures, heavy metals present, and duration of time the populations have been exposed. In the present study, after analyzing DGGE gel banding pattern and the number of bands, we found that the methanogenic diversity present in both landfills (anthropogenic system) and marshland (natural) is quite similar, except for the samples obtained from the Karimganj rice paddy field where only four bands appeared. The number of total bands observed in this study was in accordance with the number of DGGE bands reported previously [42, 51, 56, 57]. It strongly indicates that the methanogenic Archaea diversity in both landfill and marshland is influenced by sampling location rather than type.

## 4. Conclusions

In the sequencing of the molecular marker for archaeal diversity, 16S rDNA identified the orders, named as Methanobacteriales and Methanosarcinales in both landfill sites and the phylum Crenarchaeota (thermophilic) in marshland. Quantitative PCR indicated a higher abundance of methanogens in landfill compared to that of marshland sites. The knowledge about the composition and abundance of methanogenic Archaea in a landfill may provide information on the decomposition mechanism of municipal solid waste and the subsequent generation of methane. This information can be exploited for controlling methane emission from landfill by mitigation process. The increasing knowledge about the genomic content of microbes belonging to the phylum Thaumarchaeota (mesophilic) will enrich our understanding of their adaptative behavior in the transposition from thermophily to mesophily. This indicates whether they follow a similar or different evolutionary pattern with respect to the phylum, Euryarchaeota.

## Figures and Tables

**Table 1:** Sampling point from Delhi landfill site (Ghazipur, Bhalswa, and Okhla) and Southern Assam marshland (Silcoorie Lake (Silchar), Badarpur, and Karimganj) areas.

Feature	Ghazipur	Bhalswa	Okhla	Silcoorie Lake (Silchar)	Badarpur	Karimganj
Location	28°37′22.4′′N 77°19′25.7′′E	28°44′27.16′′N 77.9°9′27.92′′E	28°30′42′′N 77°16′59′′E	24°45′178′′N 92°46′58.3′′E	24°54′00′′N 92°36′00′′E	24°52′00′′N 92°21′00′′E

Type	Leachate	Soil and leachate	Leachate	Lake sediment	Marshy pond	Rice paddy

Depth	150 cm	200 cm	150 cm	40 cm	100 cm	Surface

**Table 2:** Chemical analysis of leachate samples obtained from three landfill and marshland sites. All parameters are in mg L^−1^ adapted from Ghosh et al. 2015 and Roy and Gupta 2012 [37, 38].

Parameter	Bhalswa	Ghazipur	Okhla	Silcoorie Lake (Silchar)	Karimganj	Badarpur
pH	8.1	8.4	8.3	6.27	6.89	6.69
TDS	31,469	29,700	33,657	53,282	68,293	65,312
COD	29,930	31,600	29,020	NA	NA	NA
Fe	10.32	9.81	6.51	2.81	6.17	3.89
Cl	227	1174.2	264	9.11	12.60	16.31

**Table 3:** List of primers for PCR amplification of 16S rDNA gene and DGGE used in the present study.

Primer	Sequence (5′-3′)	Reference
MET86 F	GCT CAG TAA CAC GTG	Wright and Pimm 2003 [36]
MET1340 R	CGG TGT GTG CAA GGA	

519FWD	CAGCCGCCGCGGTAA	Cheng et al. 2009 [35]
915REV	GTGCTCCCCCGCCAATTCCT	

915GC	CGC CCG GGG CGC GCC CCG GGC GGG GCG GGG GCA CGG GGGGTT GTGCTCCCCCGCCAATTCCT	Cheng et al. 2009 [35]

**Table 4:** List of accession numbers of the sequences submitted in NCBI and their percent similarity with database along with the sampling sites.

Accession number	Sample ID	Tentative organism name	Location
KM041239.1	MET1 LAND	*Methanoculleus thermophiles* (99% similarity with JF330114.1)	Bhalswa landfill
KM041240.1	MET2 LAND	*Methanoculleus thermophiles *(99% similarity with JF330114.1)	Bhalswa landfill
KM041241.1	MET3 LAND	Uncultured archaeon clone (99% similarity with AB535355.1)	Bhalswa landfill
KM041242.1	METG1 LAND	Uncultured archaeon clone (94% similarity with JF807145.1)	Ghazipur landfill
KM041248.1	METK2 MARSH	Uncultured euryarchaeote clone (98% similarity with KF360011.1)	Karimganj
KM041249.1	METK4 MARSH	Uncultured archaeon clone (100% similarity with JQ245687.1)	Karimganj
KM041250.1	SD1 MARSH	Uncultured archaeon clone (97% similarity with JF304136.1)	Silcoorie Lake (Silchar)
KM041251.1	SD3 MARSH	Uncultured archaeon clone (97% similarity with JF708703.1)	Silcoorie Lake (Silchar)
KM041252.1	SD4 MARSH	Uncultured archaeon clone (91% similarity with AB364893.1)	Silcoorie Lake (Silchar)
KM041243.1	MetG2 landfill	*Methylobacillus flagellates *(97% similarity with NR_074178.1)	Ghazipur landfill
KM041244.1	MetG3 landfill	*Methylobacillus arboreus *(99% similarity with NR_108851.1)	Ghazipur landfill
KM041245.1	MEtG4 landfill	*Methylobacillus flagellates *(99% similarity with NR_074178.1)	Ghazipur landfill
KM041246.1	MetG6 landfill	*Methylobacillus flagellates *(98% similarity with NR_074178.1)	Ghazipur landfill
KM041247.1	MetG7 landfill	*Methylobacillus arboreus *(99% similarity with NR_108851.1)	Ghazipur landfill

**Table 5:** Copy number of methanogens present per gram samples of Okhla and Bhalswa landfill site, Delhi, and Silcoorie Lake, Assam, India.

Pure culture	Okhla	Bhalswa	Silcoorie Lake (Silchar)
*M. arbophilicus*	3.98*e* + 014–6.8*e* + 014	5.38*e* + 014–9.7*e* + 014	2.78*e* + 011–7.8*e* + 011
*M. bryantii*	1.14*e* + 017–1.8*e* + 017	4.6*e* + 016–8.2*e* + 016	1.2*e* + 013–2.1*e* + 013
*M. mobile*	6.67*e* + 015–7.5*e* + 015	3.42*e* + 015–4.8*e* + 015	3.97*e* + 012–4.1*e* + 012
*M. mazei*	1.89*e* + 014–2.5*e* + 014	2.21*e* + 015–2.8*e* + 015	1.13*e* + 012–1.5*e* + 012

**Table 6:** Methanogenic pathways and microorganisms that are associated.

Domain: Archaea; kingdom: Archaebacteria; phylum: Euryarchaeota		
Methanogenic pathway	Orders	Reaction
Acetoclastic	Methanosarcinales	CH_3_COOH→CH_4_ + CO_2_
Hydrogenotrophic	Methanosarcinales	4H_2_ + CO_2_→CH_4_ + 2H_2_O
	Methanobacteriales	4HCOOH→CH_4_ + 3CO_2_ + 2H_2_O
	Methanococcales	
	Methanomicrobiales	
	Methanopyrales	
Methylotrophic	Methanosarcinales	4CH_3_OH→3CH_4_ + CO_2_ + 2H_2_O

## Abbreviations

MSW: Municipal solid waste
GHGs: Green House Gases
DGGE: Denaturing gradient gel electrophoresis
mM: Millimolar
Mg: Milligram
mL: Millilitre
Tg: Teragram.

## References

[1] Yvon-Durocher G., Allen A. P., Bastviken D. (2014). Methane fluxes show consistent temperature dependence across microbial to ecosystem scales. *Nature*.

[2] Bridgham S. D., Cadillo-Quiroz H., Keller J. K., Zhuang Q. (2013). Methane emissions from wetlands: biogeochemical, microbial, and modeling perspectives from local to global scales. *Global Change Biology*.

[3] Houghton J. T., Ding Y., Griggs D. J. (2001). *Climate Change 2001: The Scientific Basis*.

[4] Solomon S. (2007). *Climate Change 2007—The Physical Science Basis: Working Group I Contribution to the Fourth Assessment Report of the IPCC*.

[5] Angel R., Claus P., Conrad R. (2012). Methanogenic archaea are globally ubiquitous in aerated soils and become active under wet anoxic conditions. *The ISME Journal*.

[6] Daquiado A. R., Cho K. M., Kim T. Y., Kim S. C., Chang H.-H., Lee Y. B. (2014). Methanogenic archaea diversity in Hanwoo (*Bos taurus coreanae*) rumen fluid, rectal dung, and barn floor manure using a culture-independent method based on *mcrA* gene sequences. *Anaerobe*.

[7] Oremland R. S. (1988). Biogeochemistry of methanogenic bacteria. *Biology of Anaerobic Microorganisms*.

[8] Zinder S. H. (1993). Physiological ecology of methanogens. *Methanogenesis*.

[9] Garcia J.-L., Patel B. K. C., Ollivier B. (2000). Taxonomic, phylogenetic, and ecological diversity of methanogenic *Archaea*. *Anaerobe*.

[10] Bapteste É., Brochier C., Boucher Y. (2005). Higher-level classification of the Archaea: evolution of methanogenesis and methanogens. *Archaea*.

[11] Reeve J. N., Nölling J., Morgan R. M., Smith D. R. (1997). Methanogenesis: genes, genomes, and who's on first?. *Journal of Bacteriology*.

[12] Thauer R. K., Kaster A.-K., Seedorf H., Buckel W., Hedderich R. (2008). Methanogenic archaea: ecologically relevant differences in energy conservation. *Nature Reviews Microbiology*.

[13] Mitigation C. C. (2011). *IPCC Special Report on Renewable Energy Sources and Climate Change Mitigation*.

[14] Mayumi D., Dolfing J., Sakata S. (2013). Carbon dioxide concentration dictates alternative methanogenic pathways in oil reservoirs. *Nature Communications*.

[15] Gupta S., Choudhary N., Alappat B. J. Bioreactor landfill for MSW disposal in Delhi.

[16] Rawat M., Ramanathan A. (2011). Assessment of methane flux from municipal solid waste (MSW) landfill areas of Delhi, India. *Journal of Environmental Protection*.

[17] Talyan V., Dahiya R. P., Anand S., Sreekrishnan T. R. (2007). Quantification of methane emission from municipal solid waste disposal in Delhi. *Resources, Conservation and Recycling*.

[18] Hansen L. B., Finster K., Fossing H., Iversen N. (1998). Anaerobic methane oxidation in sulfate depleted sediments: effects of sulfate and molybdate additions. *Aquatic Microbial Ecology*.

[19] Bloom A. A., Palmer P. I., Fraser A., David S. R., Frankenberg C. (2010). Large-scale controls of methanogenesis inferred from methane and gravity spaceborne data. *Science*.

[20] Singh V., Mittal A. Toxicity analysis and public health aspects of municipal landfill leachate: a case study of Okhla Landfill, Delhi.

[21] Liu D., Ding W., Jia Z., Cai Z. (2012). The impact of dissolved organic carbon on the spatial variability of methanogenic archaea communities in natural wetland ecosystems across China. *Applied Microbiology and Biotechnology*.

[22] Walter B. P., Heimann M., Shannon R. D., White J. R. (1996). A process-based model to derive methane emissions from natural wetlands. *Geophysical Research Letters*.

[23] Chasar L. S., Chanton J. P., Glaser P. H., Siegel D. I. (2000). Methane concentration and stable isotope distribution as evidence of rhizospheric processes: Comparison of a fen and bog in the glacial Lake Agassiz Peatland complex. *Annals of Botany*.

[24] Avery G. B., Shannon R. D., White J. R., Martens C. S., Alperin M. J. (2003). Controls on methane production in a tidal freshwater estuary and a peatland: methane production via acetate fermentation and CO_2_ reduction. *Biogeochemistry*.

[25] Huang L.-N., Chen Y.-Q., Zhou H., Luo S., Lan C.-Y., Qu L.-H. (2003). Characterization of methanogenic Archaea in the leachate of a closed municipal solid waste landfill. *FEMS Microbiology Ecology*.

[26] Laloui-Carpentier W., Li T., Vigneron V., Mazéas L., Bouchez T. (2006). Methanogenic diversity and activity in municipal solid waste landfill leachates. *Antonie van Leeuwenhoek*.

[27] Steinberg L. M., Regan J. M. (2008). Phylogenetic comparison of the methanogenic communities from an acidic, oligotrophic fen and an anaerobic digester treating municipal wastewater sludge. *Applied and Environmental Microbiology*.

[28] Ban Q., Li J., Zhang L., Zhang Y., Jha A. K. (2013). Phylogenetic diversity of methanogenic archaea and kinetics of methane production at slightly acidic conditions of an anaerobic sludge. *International Journal of Agriculture and Biology*.

[29] Tringe S. G., Rubin E. M. (2005). Metagenomics: DNA sequencing of environmental samples. *Nature Reviews Genetics*.

[30] Bardgett R. D., van der Putten W. H. (2014). Belowground biodiversity and ecosystem functioning. *Nature*.

[31] Pace N. R. (1996). New perspective on the natural microbial world: molecular microbial ecology. *ASM News*.

[32] Kirk J. L., Beaudette L. A., Hart M. (2004). Methods of studying soil microbial diversity. *Journal of Microbiological Methods*.

[33] Amann R. I., Ludwig W., Schleifer K.-H. (1995). Phylogenetic identification and in situ detection of individual microbial cells without cultivation. *Microbiological Reviews*.

[34] Keller M., Zengler K. (2004). Tapping into microbial diversity. *Nature Reviews Microbiology*.

[35] Cheng Y. F., Mao S. Y., Liu J. X., Zhu W. Y. (2009). Molecular diversity analysis of rumen methanogenic Archaea from goat in eastern China by DGGE methods using different primer pairs. *Letters in Applied Microbiology*.

[36] Wright A.-D. G., Pimm C. (2003). Improved strategy for presumptive identification of methanogens using 16S riboprinting. *Journal of Microbiological Methods*.

[37] Ghosh P., Gupta A., Thakur I. S. (2015). Combined chemical and toxicological evaluation of leachate from municipal solid waste landfill sites of Delhi, India. *Environmental Science and Pollution Research*.

[38] Roy S., Gupta A. (2012). Water quality assessment of river barak and tributaries in Assam, India. *Pollution Research*.

[39] Altschul S. F., Gish W., Miller W., Myers E. W., Lipman D. J. (1990). Basic local alignment search tool. *Journal of Molecular Biology*.

[40] Tamura K., Stecher G., Peterson D., Filipski A., Kumar S. (2013). MEGA6: molecular evolutionary genetics analysis version 6.0. *Molecular Biology and Evolution*.

[41] Kimura M. (1980). A simple method for estimating evolutionary rates of base substitutions through comparative studies of nucleotide sequences. *Journal of Molecular Evolution*.

[42] Watanabe T., Kimura M., Asakawa S. (2010). Diversity of methanogenic archaeal communities in Japanese paddy field ecosystem, estimated by denaturing gradient gel electrophoresis. *Biology and Fertility of Soils*.

[43] Borrel G., Harris H. M. B., Tottey W. (2012). Genome sequence of ‘*Candidatus* Methanomethylophilus alvus’ Mx1201, a methanogenic archaeon from the human gut belonging to a seventh order of methanogens. *Journal of Bacteriology*.

[44] Juottonen H. (2008). *Archaea, Bacteria, and methane production along environmental gradients in fens and bogs [Ph.D. thesis]*.

[45] Borrel G., O'Toole P. W., Harris H. M. B., Peyret P., Brugère J.-F., Gribaldo S. (2013). Phylogenomic data support a seventh order of methylotrophic methanogens and provide insights into the evolution of methanogenesis. *Genome Biology and Evolution*.

[46] Iverson V., Morris R. M., Frazar C. D., Berthiaume C. T., Morales R. L., Armbrust E. V. (2012). Untangling genomes from metagenomes: revealing an uncultured class of marine euryarchaeota. *Science*.

[47] Lloyd K. G., Schreiber L., Petersen D. G. (2013). Predominant archaea in marine sediments degrade detrital proteins. *Nature*.

[48] Anderson I., Wirth R., Lucas S. (2011). Complete genome sequence of *Staphylothermus hellenicus*P8^T^. *Standards in Genomic Sciences*.

[49] Brochier-Armanet C., Gribaldo S., Forterre P. (2012). Spotlight on the thaumarchaeota. *The ISME Journal*.

[50] Galand P. E., Fritze H., Conrad R., Yrjälä K. (2005). Pathways for methanogenesis and diversity of methanogenic archaea in three boreal peatland ecosystems. *Applied and Environmental Microbiology*.

[51] Ikenaga M., Asakawa S., Muraoka Y., Kimura M. (2004). Methanogenic archaeal communities in rice roots grown in flooded soil pots: estimation by PCR-DGGE and sequence analyses. *Soil Science and Plant Nutrition*.

[52] Spang A., Poehlein A., Offre P. (2012). The genome of the ammonia-oxidizing *Candidatus* nitrososphaera gargensis: insights into metabolic versatility and environmental adaptations. *Environmental Microbiology*.

[53] Chaudhary P. P., Brablcová L., Buriánková I., Rulík M. (2013). Molecular diversity and tools for deciphering the methanogen community structure and diversity in freshwater sediments. *Applied Microbiology and Biotechnology*.

[54] Piterina A. V., Pembroke J. T. (2013). Use of PCR-DGGE based molecular methods to analyse microbial community diversity and stability during the thermophilic stages of an ATAD wastewater sludge treatment process as an aid to performance monitoring. *ISRN Biotechnology*.

[55] Watanabe T., Kimura M., Asakawa S. (2006). Community structure of methanogenic archaea in paddy field soil under double cropping (rice-wheat). *Soil Biology and Biochemistry*.

[56] Watanabe T., Kimura M., Asakawa S. (2007). Dynamics of methanogenic archaeal communities based on rRNA analysis and their relation to methanogenic activity in Japanese paddy field soils. *Soil Biology and Biochemistry*.

[57] Brablcová L., Buriánková I., Badurová P., Chaudhary P. P., Rulík M. (2015). Methanogenic archaea diversity in hyporheic sediments of a small lowland stream. *Anaerobe*.

[58] Mach V., Blaser M. B., Claus P., Chaudhary P. P., RulÃ­k M. (2015). Methane production potentials, pathways, and communities of methanogens in vertical sediment profiles of river Sitka. *Frontiers in Microbiology*.

[59] Muyzer G. (1999). DGGE/TGGE a method for identifying genes from natural ecosystems. *Current Opinion in Microbiology*.

[60] Muyzer G., De Waal E. C., Uitterlinden A. G. (1993). Profiling of complex microbial populations by denaturing gradient gel electrophoresis analysis of polymerase chain reaction-amplified genes coding for 16S rRNA. *Applied and Environmental Microbiology*.

[61] Macnaughton S. J., Stephen J. R., Venosa A. D., Davis G. A., Chang Y.-J., White D. C. (1999). Microbial population changes during bioremediation of an experimental oil spill. *Applied and Environmental Microbiology*.

[62] Yu Y., Lee C., Kim J., Hwang S. (2005). Group-specific primer and probe sets to detect methanogenic communities using quantitative real-time polymerase chain reaction. *Biotechnology and Bioengineering*.

